# First-in-Human ^212^Pb-PSMA–Targeted α-Therapy SPECT/CT Imaging in a Patient with Metastatic Castration-Resistant Prostate Cancer

**DOI:** 10.2967/jnumed.123.267189

**Published:** 2024-04

**Authors:** Matthew R. Griffiths, David A. Pattison, Melissa Latter, Kevin Kuan, Stephen Taylor, William Tieu, Thomas Kryza, Danielle Meyrick, Boon Quan Lee, Aaron Hansen, Stephen E. Rose, Simon G. Puttick

**Affiliations:** 1 Department of Nuclear Medicine and Specialist PET Services, Royal Brisbane and Women’s Hospital, Brisbane, Queensland, Australia;; 2 AdvanCell, Sydney, New South Wales, Australia; and; 3 Department of Medical Oncology, Princess Alexandra Hospital, Brisbane, Queensland, Australia

There is significant interest in the development of ^212^Pb-PSMA–based targeted α-therapy for patients with metastatic castration-resistant prostate cancer. A previous phantom study has shown that ^212^Pb SPECT is feasible by imaging the 238.6 keV and 75 to 91 keV γ-emissions produced after the β-decay of ^212^Pb to its α-emitting progeny (1).

Here we present—to the best of our knowledge—the first human ^212^Pb SPECT/CT images published to date. They were acquired after administration of 60 MBq of ^212^Pb-ADVC001 to a 73-y-old man with metastatic castration-resistant prostate cancer. This study was approved by the local institutional review board. Imaging was at 1.5, 5, 20, and 28 h after infusion. Two simultaneous triple-energy window acquisitions (78 keV ± 20% with 20% scatter [31% abundance] and 239 keV ± 10% with 10% scatter [43% abundance] were obtained using a Siemens Intevo Bold (high-energy collimators at 30 s per view for 120 views per rotation at 2 bed positions with noncircular orbits; total time, 60 min). Each energy window was reconstructed independently, and the resulting images were summed with removal of Compton-based orbit artifacts.

Representative ^212^Pb SPECT/CT images (Fig. 1) showed rapid tumor uptake of ^212^Pb-ADVC001 highly concordant with tumor burden delineated on the pretreatment ^18^F-DCFPyl PET/CT images. Images acquired after 20 h showed persistent tumor uptake despite low counts due to ^212^Pb decay (10.6 h half-life).

**FIGURE 1.**
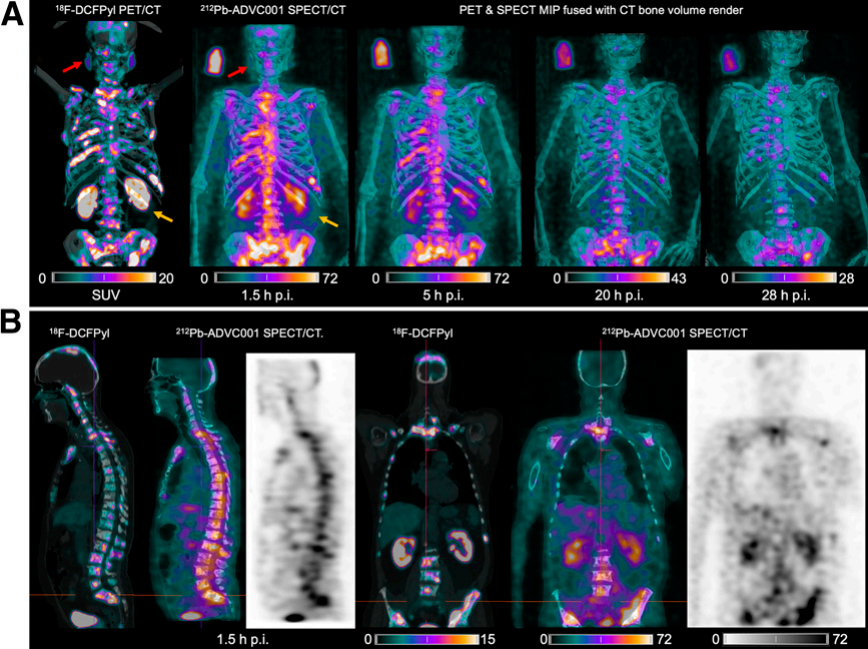
(A) ^18^F-DCFPyl PET/CT and ^212^Pb SPECT/CT images showing concordant tumor biodistribution with low salivary gland uptake (red arrow) and rapid kidney clearance of 60 MBq of ^212^Pb-ADVC001 (structure of ^212^Pb-ADVC001 available as supplemental material at http://jnm.snmjournals.org). A 3-MBq standard solution (100 mL) was included. (B) Sagittal and coronal images at 1.5 h after injection (p.i.). MIP = maximum-intensity projection.

^212^Pb is a challenging isotope to image because of the high-energy γ-rays from the lead progeny generating Compton scatter from the patient and collimator (1). Our approach of summing images reconstructed from both energy windows shows the feasibility and benefit of ^212^Pb SPECT/CT imaging in providing postinfusion radiopharmaceutical biodistribution and patient-specific dosimetry for clinical development of ^212^Pb-targeted α-therapy.

## DISCLOSURE

This work was financially supported by AdvanCell through the TheraPb clinical trial (NCT05720130). Kevin Kuan, Stephen Taylor, William Tieu, Thomas Kryza, Stephen Rose, and Simon Puttick are AdvanCell employees. Danielle Meyrick and Boon Quan Lee are AdvanCell consultants. No other potential conflict of interest relevant to this article was reported.
